# Vector competence of the African argasid tick *Ornithodoros moubata* for the Q fever agent *Coxiella burnetii*

**DOI:** 10.1371/journal.pntd.0009008

**Published:** 2021-01-06

**Authors:** Marie Buysse, Maxime Duhayon, Franck Cantet, Matteo Bonazzi, Olivier Duron

**Affiliations:** 1 MIVEGEC (Maladies Infectieuses et Vecteurs: Ecologie, Génétique, Evolution et Contrôle), Centre National de la Recherche Scientifique (CNRS), Institut pour la Recherche et le Développement (IRD), Université de Montpellier (UM), Montpellier, France; 2 CREES (Centre de Recherche en Écologie et Évolution de la Santé), Montpellier, France; 3 ASTRE, University of Montpellier, CIRAD, INRAE, Montpellier, France; 4 CIRAD, UMR ASTRE, 34398 Montpellier, France; 5 IRIM, CNRS, Université de Montpellier, Montpellier, France; Chengde Medical University, CHINA

## Abstract

Q fever is a widespread zoonotic disease caused by the intracellular bacterium *Coxiella burnetii*. While transmission is primarily but not exclusively airborne, ticks are usually thought to act as vectors on the basis of early microscopy studies. However, recent observations revealed that endosymbionts of ticks have been commonly misidentified as *C*. *burnetii*, calling the importance of tick-borne transmission into question. In this study, we re-evaluated the vector competence of the African soft tick *Ornithodoros moubata* for an avirulent strain of *C*. *burnetii*. To this end, we used an artificial feeding system to initiate infection of ticks, specific molecular tools to monitor further infections, and culture assays in axenic and cell media to check for the viability of *C*. *burnetii* excreted by ticks. We observed typical traits associated with vector competence: The exposure to an infected blood meal resulted in viable and persistent infections in ticks, trans-stadial transmissions of infection from nymphs to adults and the ability of adult ticks to transmit infectious *C*. *burnetii*. However, in contrast to early studies, we found that infection differed substantially between tick organs. In addition, while adult female ticks were infected, we did not observe *C*. *burnetii* in eggs, suggesting that transovarial transmission is not effective. Finally, we detected only a sporadic presence of *C*. *burnetii* DNA in tick faeces, but no living bacterium was further isolated in culture assays, suggesting that excretion in faeces is not a common mode of transmission in *O*. *moubata*.

## Author summary

The intracellular bacterium *Coxiella burnetii* is the agent of Q fever, a widespread zoonotic disease. Some early detection reports and microscopy studies identified ticks as vectors of Q fever but more recent studies and molecular analyses revealed that endosymbionts of ticks have been commonly misidentified as *C*. *burnetii*: It raises questions of whether ticks play an important role in Q fever transmission. In our study, we therefore experimentally re-evaluate the vector competence of the African soft tick *Ornithodoros moubata* for *C*. *burnetii*. We found that *O*. *moubata* can be infected by *C*. *burnetii* after the exposure to an infected blood meal. It resulted in viable and persistent infections in ticks, a trans-stadial transmission and the ability of adult ticks to transmit infection when feeding. Infection was however not transmitted transovarially or by faeces as early reported. Overall, we conclude that *O*. *moubata* may act as a driver of the transmission and of the spatial dispersal of Q fever among vertebrates where this tick is present in Africa.

## Introduction

Q fever (also termed coxiellosis in animals) is a widespread zoonotic disease caused by the intracellular bacterium *Coxiella burnetii* (Legionellales: Coxiellaceae) [1,2]. Cattle, sheep, and goats are most commonly infected and constitute the main reservoirs of *C*. *burnetii* [3,4]. Infection is also consistently detected in wild fauna, including capybara and sloth that may represent important additional reservoirs in remote tropical regions [5–7]. In animals, infections are usually asymptomatic and are not considered to be a veterinary problem, except in ruminants where coxiellosis is a major cause of abortion and reproductive disorders [4,8]. In humans, *C*. *burnetii* infections vary from self-limiting to severe [1,2,9]. The acute form of Q fever ranges from causing mild flu-like symptoms to pneumonia or hepatitis, which may require hospitalization. The disease can become chronic and result in premature birth or abortion, chronic fatigue syndrome, endocarditis, as well as aneurysmal, valvular or vascular infections. Sporadic cases in humans occur annually worldwide, but occasional outbreaks are also common [10–12].

Q fever is mainly an airborne zoonosis [13,14]. The common routes of infection are inhalation of contaminated barnyard dust and contact with excreta of infected animals such as birth products, urine, faeces and milk, which all harbour high titres of *C*. *burnetii*. The bacterium naturally produces spore-like small-cell variants that are able to resist extreme environmental conditions and are thus more likely to persist in the environment for long periods of time [13,14]. Spore-like small-cell variants remain highly infectious in aerosols in a wide range of temperatures, leading to the classification of *C*. *burnetii* as a category B aerosolized biological weapon [13]. Other infection pathways, including human-to-human contact, exist but they have been observed only in rare cases [13,14].

The importance of ticks in Q fever epidemiology remains debated [15]. Major pioneering studies in the 1930s and 1940s focused on *C*. *burnetii* infections in ticks [15,16]. In 1937, one of the most virulent reference strains of *C*. *burnetii* (strain RSA 493 / Nine Mile I) was isolated from a laboratory guinea pig on which field-collected Rocky Mountain wood ticks *Dermacentor andersoni* had fed [16]. At the end of the 1940s, at least seven hard and soft tick species were formally identified as competent vectors of *C*. *burnetii*: These ticks were able to acquire the bacterium from an infected animal, to maintain infection through trans-stadial transmission from larvae to nymphs or from nymphs to adults, and to transmit infectious *C*. *burnetii* to an uninfected animal (reviewed in [15]). In these pioneering studies, *C*. *burnetii* was detected in all major organs, including the midgut, salivary glands, Malpighian tubules, haemolymph, and ovaries, and such a pattern was interpreted as a systemic infection with a transmission through tick bites [15]. Large numbers of *C*. *burnetii* have been also detected in the body fluids and faeces excreted by ticks, suggesting an additional potential risk posed by tick excreta, through inhalation or direct contact [17]. Other early observations showed that *C*. *burnetii* could be maintained by ticks across several generations through transovarial transmission (i.e. from an infected female tick to its offspring) and without needing to infect vertebrates [18–20]. Altogether these case studies suggest that Q fever may be a major tick-borne zoonosis, and today many works in the field largely relate to these past studies [15]. However, recent observations, based on advances in molecular and cell biology, called some of these early results into question [15,21].

Until the late 1990s, *C*. *burnetii* was essentially screened in ticks using morphological observations, staining, and immunodetection techniques because this intracellular bacterium was notoriously difficult to culture [15,16]. However, in the 2000s, advances in molecular biology and DNA sequencing showed that ticks commonly harbour *Coxiella*-like endosymbionts (*Coxiella*-LE hereafter) that are closely related but genetically distinct to *C*. *burnetii* [22–26]. These *Coxiella*-LE are almost exclusively confined to ticks and, according to current knowledge, they pose a much lower infection risk to vertebrates compared with *C*. *burnetii* [15,21]. Extensive molecular surveys have consistently revealed that *Coxiella*-LE, and not *C*. *burnetii*, predominate in most tick species investigated thus far, with at least two thirds of tick species being naturally infected [22,24–28]. Most importantly, *Coxiella*-LE have been commonly misidentified as *C*. *burnetii*: The historic and dogmatic assertion that over 40 tick species are infected by *C*. *burnetii* (as deducted from early microscopic surveys) has been re-evaluated and most of the *Coxiella* strains initially visually identified as *C*. *burnetii* could be reclassified as *Coxiella*-LE [15]. The case studies on vector competence of ticks, their systemic infections by *C*. *burnetii* and transovarial transmission have become unreliable: Recent studies using DNA-based methods have found that the tick species used in the old literature actually harbour *Coxiella*-LE, or in certain cases other intracellular bacteria that may have been misidentified as *C*. *burnetii* [15,21]. However, the risk of misidentification still exist: several *C*. *burnetii* detection methods are in use, but many are not efficient enough to clearly distinguish between *C*. *burnetii* and *Coxiella*-LE [15,29,30]. Over the last decades, many studies aiming to estimate *C*. *burnetii* prevalence in ticks have used ambiguous typing methods, and may have continued to misidentify *Coxiella*-LE as *C*. *burnetii* [15]. To date, only few studies produced clear evidence that field ticks are infected by *C*. *burnetii*[15].

Today, ticks are still the focus of many field studies of Q fever epidemiology. There is no doubt that ticks may be infected by *C*. *burnetii* in nature since several strains have been successfully isolated from wild ticks [15]. In recent years, remarkable progress has been made in designing new molecular techniques to detect *C*. *burnetii*, but with variable success [15]. Some techniques, based on DNA sequencing, allow for the unambiguous distinction between *C*. *burnetii* and *Coxiella*-LE [25,27], but others, such as those based on the detection of the IS1111 genetic element (present in *C*. *burnetii* and some *Coxiella*-LE), are not reliable for diagnosis in ticks [29,30]. Only few recent studies have produced clear and unambiguous evidence that ticks can carry *C*. *burnetii* [15]. In the noteworthy case study by Pacheco et al. [31], a natural *C*. *burnetii* infection in *Amblyomma* spp. ticks was confirmed through diverse detection methods, including haemolymph tests, isolation in Vero cells, and multilocus DNA sequencing. In a recent experimental infection study, Körner et al. [32] showed that the European hard ticks *Ixodes ricinus* and *Dermacentor marginatus* can be infected following an infected blood meal and further excrete infectious *C*. *burnetii* in faeces. However, *C*. *burnetii* was not detected in the blood used for feeding of infected *I*. *ricinus* and *D*. *marginatus* specimens [32]. This notably suggests that transmission by ticks could occur by inhalation of faeces containing infectious *C*. *burnetii* rather than by tick bites.

In this study, we re-evaluate the vector competence of the African soft tick *Ornithodoros moubata* for an avirulent strain of *C*. *burnetii*. This tick species is widespread in East Africa and is the vector of relapsing fever in humans and of African swine fever in pigs. In this region, the presence of Q fever has been known for over 60 years, where it remains a neglected zoonosis [33,34]. Most notably, the transmission routes have not been clearly established, while Q fever consistently accounts for cases of human febrile illness and infective endocarditis in Africa [33,34]. In an early study published in 1943, *O*. *moubata* was found to transmit infectious *C*. *burnetii* to Guiana pigs through biting [20]. Recent surveys showed that *O*. *moubata* was infected not by *Coxiella*-LE, but by an intracellular bacterium, a *Francisella*-like endosymbiont (*Francisella*-LE; Thiotrichales: Francisellaceae) [35]. This endosymbiont is an obligate nutritional mutualist required for survival and reproduction of *O*. *moubata* since it synthesizes B vitamins that are deficient in the blood meal of ticks [35]. *Francisella*-LE is naturally present in all *O*. *moubata* specimens and maternally transmitted to all maturing tick oocytes [35]. Here, we experimentally examined the ability of *O*. *moubata* to acquire *C*. *burnetii* from an infected blood meal, the trans-stadial transmission of infection from nymphs to adults and the ability to further transmit infectious *C*. *burnetii* through faeces and bites. To this aim, we combined (i) an artificial feeding system to infect ticks in controlled experimental conditions with (ii) specific molecular tools to quantify *C*. *burnetii* infections and to avoid misidentification with other microorganisms present in ticks and with (iii) axenic and cellular cultures to check for the viability of *C*. *burnetii* excreted by ticks. We also examined the molting and mortality rates of ticks to assess whether *C*. *burnetii* infection is deleterious to their vectors. We further characterized the abundance of *C*. *burnetii* within the tick tissues, and investigated whether the infection could be transovarially transmitted through tick generations.

## Methods

### Ethics statement

Tick feeding and manipulation were performed in a Biosafety Level 2 insectarium according to the regulations established by the Ethical and Animal Welfare Committee of the institution where the experiments were conducted (CIRAD, Montpellier, France), complying with the European legislation. During the experiment, blood was taken from cows sheltered in the CIRAD animal facility according to a protocol approved under number APAFIS#1445-2015081217184829v2 by the French Ministry of Research. All cultivations of *C*. *burnetii* were carried out under Biosafety Level 2 laboratory conditions at IRIM (Montpellier, France).

### Ticks and housing conditions

All ticks used in this study came from an *O*. *moubata sensu stricto* laboratory colony (Neuchâtel strain) initiated from field specimens collected in Southern Africa and maintained in the CIRAD insectary, Montpellier, France [35]. Ticks were maintained in the laboratory at 26°C with 80–90% relative humidity under complete darkness. The life cycle of *O*. *moubata* includes one larval stage, three to five nymphal stages, and the adult stage with a longevity of several years. A blood meal made of heparinized cow blood was offered to ticks at least every 7 weeks using an artificial feeding system as follows: Ticks were allowed to feed on blood through a parafilm membrane using a specific apparatus including three parts: (i) A tick chamber closed on top by a nylon cloth to avoid tick escape and closed below by the parafilm membrane, (ii) a blood chamber containing a magnet, and (iii) a hot magnetic steering device to mix and warm blood at 38°C. With this method, all the ticks used in the present experiments successfully fed at all stages. After feeding, each batch of ticks was kept in separate plastic containers until the next feeding.

### Cultivation of *Coxiella burnetii*

We used a transposon mutant of Nile Mile RSA439 (phase II, clone 4) (NMIIC4) of *C*. *burnetii* [36]. This strain is avirulent in mammalian but not in arthropod models as further described. This phase II *C*. *burnetii* clone primarily originates from spontaneous mutations after several *in vitro* passages of extremely virulent phase I *C*. *burnetii* and present a truncated lipopolysaccharide (LPS) [37]. These non-reversible mutations result in a strong attenuation of virulence but phase II *C*. *burnetii* are internalized more efficiently than phase I organisms by host cells and replicate then with similar kinetics than phase I [37]. The phase II *C*. *burnetii* remains yet virulent to some arthropods as it kills larvae of the greater wax moth, *Galleria mellonella* [36]. The NMIIC4 clone has been authorized for biosafety level 2 (BSL-2) manipulation and represents an convenient model to study *C*. *burnetii* infections [36].

We used in this study an eGFP-expressing, chloramphenicol-resistant NMIIC4 *C*. *burnetii* strain (Tn1832 strain) previously obtained by transposon insertion [36]. The transposon insertion is located at bp 1,776,921 of *C*. *burnetii* NMII genome, corresponding to an intergenic region between the genes *cbu1847b* and *cbu1849* (encoding for hypothetical proteins) and does not disrupt any bacterial function [36]. This *C*. *burnetii* strain was cultivated in the axenic medium ACCM2 supplemented with chloramphenicol (3 μg/ml) and incubated in a humidified atmosphere of 5% CO_2_ and 2.5% O_2_ at 37°C. Living *C*. *burnetii* obtained from this axenic medium (ACCM2) culture were used to initiate infections in ticks.

### *Coxiella burnetii* infections in ticks and post-infection rearing

Fourth-instar nymphs of *O*. *moubata* were randomly allocated to one of two batches: one exposed to infectious *C*. *burnetii* (infected batch), and another not exposed to *C*. *burnetii* (control batch). All specimens were subsequently fed on the same heparinized cow blood pack using an artificial feeding system. For the infected treatment batch, living *C*. *burnetii* obtained from an axenic culture were added to the blood meal at a final concentration of 5.10^−4^ bacteria/mL. For the control batch, nothing was added to the blood meal. After feeding, each batch of ticks was kept in separate plastic containers stored under standard housing conditions until the molting of nymphs to adults. A second blood meal was then provided to a random subsample of adults from each batch. No *C*. *burnetii* was added in this second blood meal, meaning that ticks from the infected treatment batch were exposed only once to *C*. *burnetii* (i.e. during the first blood meal). Engorged adult ticks were allowed to mate, and females were further allowed to lay eggs in individual vials. Samples of all heparinized cow blood packs were examined before and after the blood meal to control for the presence of *C*. *burnetii*.

### DNA isolation and detection of *Coxiella burnetii*

DNA was extracted from blood samples and from the whole body, dissected organs, eggs and faeces of ticks using a DNA extraction kit according to the manufacturer’s instructions (QIAGEN). Before extraction, faeces were solubilized into a standardized volume of phosphate-buffered saline solution (PBS 1X). To eliminate external (i.e. cuticular) contamination, ticks and eggs tick specimens were surface cleaned with bleach before DNA extraction following an existing protocol [28]. Tick organs (salivary glands, gut, Malpighian tubule system, and ovaries/testes) were dissected with sterile blades and forceps under microscopic observation and rinsed with sterile saline solution before a transfer to the QIAGEN lysis buffer.

Real-time quantitative polymerase chain reaction (qPCR) was further used to check for the presence and the titration of *C*. *burnetii*. qPCR was performed with a LightCycler 480 (Roche) using the SYBR Select Master Mix (Thermo Scientific). For tick specimens or organs, two PCRs were performed: one was specific for the bacterial *egfp* gene, and the other was specific for the *O*. *moubata OmAct2* gene (Table 1). Assuming that both genes are present in a single copy per haploid genome of *C*. *burnetii* and *O*. *moubata*, the ratio between *egfp* and *OmAct2* concentrations provides the number of *C*. *burnetii* genomes relative to the number of *O*. *moubata* genomes, thus correcting for tick size or tick organ size. Each DNA template was analyzed in triplicate for *egfp* and *OmAct2* quantification. Standard curves were plotted using dilutions of a pEX-A2 vector (Eurofins) containing one copy of each of the *egfp* and *OmAct2* gene fragments. For faeces and blood samples, only the qPCR specific for the bacterial *egfp* gene was performed, as described above.

**Table 1:** Genes and primers used in this study. Nested PCR and qPCR assays were conducted for detection, titration, and typing of *Coxiella burnetii*.

Gene	Hypothetical product	Primers (5'-3')		Tm	Fragment size	Reference
*Ornithodoros moubata*						
*OmAct2*	Actin	Omou-actqF2	CGGTATTGCCGACCGTATGC	60°C	qPCR assay:	[35]
		Omou-actqR1	GCTGGAAGGTGGACAGGGAG		Orni_act2_qF/Orni_act2_qR: 140bp	
*Coxiella burnetii*						
*egfp*	Enhanced green	Coxbur-GFPqF2	CCTGGGGCACAAGCTGGAG	60°C	qPCR assay:	This study
	fluorescent protein	Coxbur-GFPqR2	GCTGCACGCTGCCGTCCTCG		Coxbur-GFPqF2/Coxbur-GFPqR2: 124bp	
*Coxiella*						
*rpoB*	DNA-directed RNA	CoxrpoB_F2	GGGCGNCAYGGWAAYAAAGGSGT	56°C	Nested PCR assay:	[25]
	polymerase beta chain	CoxrpoB_R1	CACCRAAHCGTTGACCRCCAAATTG		1st round PCR: CoxrpoBF2/CoxrpoBR1: 610bp	
		CoxrpoB_F3	TCGAAGAYATGCCYTATTTAGAAG		2nd round PCR: CoxrpoBF3/CoxrpoBR3: 542bp	
		CoxrpoB_R3	AGCTTTMCCACCSARGGGTTGCTG			

Independent nested PCR assays for *C*. *burnetii* identification were also performed with the same DNA templates by amplifying the *Coxiella rpoB* gene fragment using specific primers (Table 1). The first PCR round was performed in a 10-μL volume containing 20–50 ng of DNA, 1.25 mM of each dNTP (Thermo Scientific), 7.5 mM of MgCl_2_ (Roche Diagnostics), 3 μM of each external primer, 1 μL of 10X PCR buffer (Roche Diagnostics), and 0.5 U of Taq DNA polymerase (Roche Diagnostics). A 1-μL aliquot of the PCR product from the first reaction was then used as a template for the second round of amplification. The second PCR round was performed in a 25-μL volume containing 3 mM of each dNTP (Thermo Scientific), 18.75 mM of MgCl_2_ (Roche Diagnostics), 7.5 μM of each internal primer, 2.5 μL of 10X PCR buffer (Roche Diagnostics), and 1.25 U of Taq DNA polymerase (Roche Diagnostics). PCR amplifications were performed under the following conditions: initial denaturation at 84°C for 3 min, 35 cycles of denaturation (94°C, 30 s), annealing (Tm = 56°C, 30 s), extension (72°C, 1 min), and a final extension at 72°C for 3 min. Known positive and negative individuals were used as controls in each PCR assay. All PCR products were visualized via electrophoresis in a 1.5% agarose gel. Positive *rpoB* PCR products were purified and sequenced in both directions (Eurofins). Sequence chromatograms were manually cleaned with CHROMAS LITE (http://www.technelysium.com.au/chromas_lite.html), and alignments were performed using CLUSTALW, implemented in the MEGA software [38].

### Amplification of *Coxiella burnetii* from blood and infection assay

We examined the viability of *C*. *burnetii* excreted by ticks as follows. Samples were haemolysed in distilled water and 2 mL was inoculated in 10 ml of ACCM2 and incubated in a humidified atmosphere of 5% CO_2_ and 2.5% O_2_ at 37°C. One week after inoculation, samples were re-inoculated in fresh ACCM2 and the replication of eGFP-expressing *C*. *burnetii* was monitored over time for 6 days via fluorescence microscopy. Bacteria were then collected by centrifugation at 1940 x g for 45 min, resuspended in DMEM supplemented with 10% foetal calf serum and used to infect human U2OS cells (this cell line is widely used in biomedical research and has high infection efficiencies by *C*. *burnetii* [39]). Cells were centrifuged for 10 min at 110 x g and incubated in a humidified atmosphere of 5% CO_2_ at 37°C. Intracellular bacterial replication was monitored over time via fluorescence microscopy. At 7 days post-infection, cells were fixed in 4% (wt/vol) paraformaldehyde in PBS solution at room temperature for 20 min. Samples were then rinsed in PBS solution and incubated in blocking solution (0.5% BSA, 50 mM NH4Cl in PBS solution, pH 7.4). Subsequently, cells were incubated with an anti-LAMP1 antibody (Sigma) diluted in blocking solution for 1 h at room temperature, rinsed five times in PBS solution, and further incubated for 1 h with the secondary antibodies diluted in the blocking solution. Coverslips were mounted by using Fluoromount mounting medium (Sigma) supplemented with Hoechst 33258 for DNA staining. In all cases, images were acquired using an EVOS inverted fluorescence microscope (Thermo Scientific).

### Statistical analyses

Analyses were carried out using the R statistical package (v3.6.2) and the lme4 package [40]. The *C*. *burnetii* load was analyzed using linear mixed-effects models after applying a log-transformation. The proportion of infected organs was analyzed using generalized mixed-effects models and a binomial distribution. To build models, organs (midgut/salivary glands/Malpighian tubules/carcass/reproductive organs) and sex (female/male) were fitted as fixed explanatory variables, while tick specimens were fitted as a random effect. The maximal model, including all higher-order interactions, was simplified by sequentially eliminating interactions and non-significant terms to establish a minimal model. A likelihood ratio test (LRT) (using a chi-square distribution and a *p*-value cut-off of 0.05) was performed to establish the difference between sequential models. To refine analyses, post hoc analyses were performed to establish significant differences between organ types.

## Results

### *Coxiella burnetii* infections in ticks

We provided a blood meal to 30 fourth-instar nymphs of *O*. *moubata* reared in the same standard laboratory conditions. No *C*. *burnetii* DNA was detected through qPCR in the blood prior to the experiment. For 20 nymphs, *C*. *burnetii* from an axenic culture was administered with the blood meal (infected batch), while for the 10 other nymphs, no bacterium was added (control batch). All the nymphs (*n* = 30) fully engorged in 1 h and were placed in separate plastic containers. Over the next 7 weeks, the exposure to *C*. *burnetii* did not affect the survival and molting of the ticks: All the nymphs of the infected batch survived and molted to adults (*n* = 20). None showed apparent physical abnormality and disease symptoms. All the ticks of the control batch also survived and molted to adults (*n* = 10). By the seventh week, all ticks had digested a large portion of their previous blood and produced abundant faeces.

**Fig 1:**
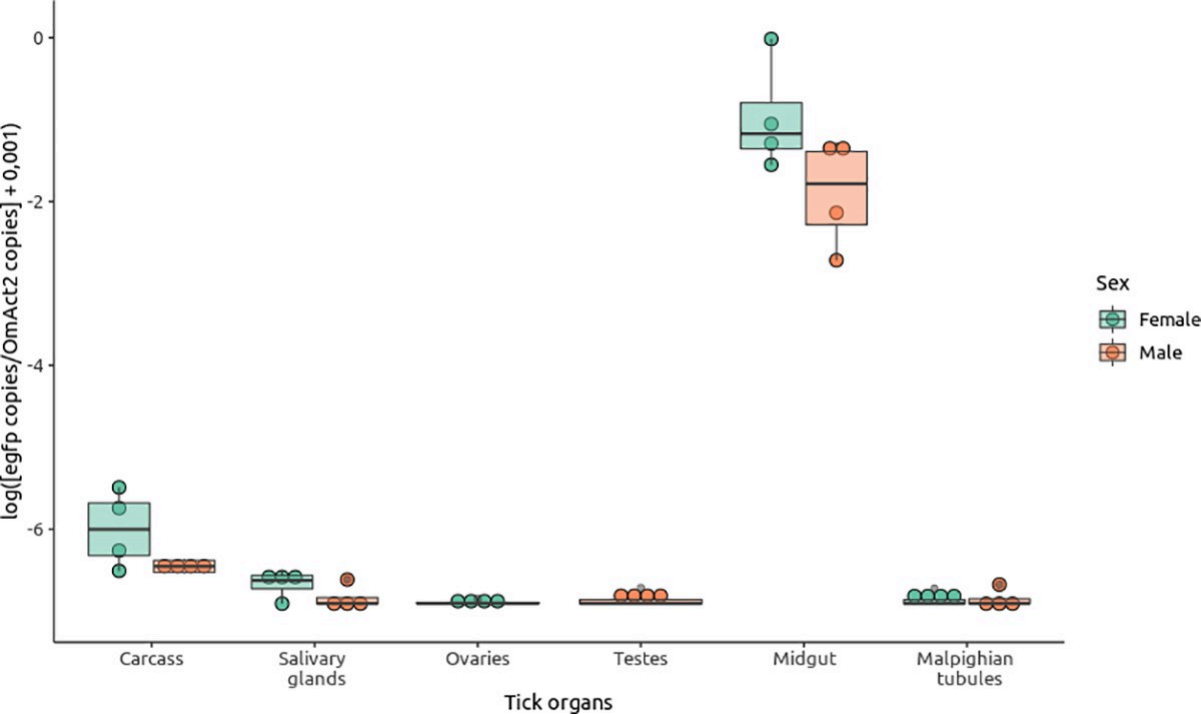
*Coxiella burnetii* according to sex and organ in *Ornithodoros moubata* ticks. Infection densities in males (*n* = 4, orange circles and boxplots) and females (*n* = 4, green circles and boxplots) were quantified by using the ratio of bacterial *egfp* gene copies per tick *OmAct2* gene copy and represented as the logarithm values (× + 0.001).

Seven weeks after feeding, a subsample of *O*. *moubata* adults (four from the control batch and eight from the infected batch; *n* = 12) were analyzed by qPCR for infection. None of the *O*. *moubata* adults from the control batch was infected by *C*. *burnetii*, whereas all of the eight adults (four females and four males) from the infected batch were infected. Interestingly, qPCR also revealed variation of *C*. *burnetii* abundance between tick tissues (Fig 1). Indeed, *C*. *burnetii* was detected in the midgut of all adults, in the salivary glands of five of eight adults, in the Malpighian tubules of two of eight adults, in the reproductive organs of two of eight adults (i.e. in the ovaries of one of four females and the testes of one of four males) and in the rest of body of all eight adults. The prevalence of *C*. *burnetii* in these organs was determined mainly by the organ type (χ^2^ = 25.237, *p* = 5.10^−5^) and was independent of specimen sex (χ^2^ = 0.7153, *p* = 0.40). When considering the bacterial load, both organ type (χ^2^ = 80.87, *p* = 2.2.10^−16^) and sex (χ^2^ = 4.0093, *p* = 0.04525) affected the density of *C*. *burnetii*. Thus, the density of *C*. *burnetii* in female organs was higher than in male organs (Fig 1). The density of *C*. *burnetii* was 277–2304 times higher in the midgut than in any of the other organs, both for male and female ticks. With the exception of the midgut, pairwise comparisons showed that the density of *C*. *burnetii* in salivary glands was 1.8–2.7 times higher than in reproductive organs and Malpighian tubules (post hoc analysis, χ^2^ = 4.0613, *p* = 0.044 and χ^2^ = 0.4643, *p* = 0.496, respectively) but 3.024 times lower than in the rest of body (post hoc analysis, χ^2^ = 6.7787, *p* = 0.009) (Fig 1).

### Excretion of *Coxiella burnetii* in tick faeces

Faeces of *O*. *moubata* were collected 7 weeks after feeding and assayed for the presence of *C*. *burnetii* (one faeces sample per tick). qPCR was negative for all samples collected from the control batch (*n* = 4). By contrast, qPCR revealed the presence of *C*. *burnetii* DNA in the faeces of two of twelve adults. The number of *C*. *burnetii* DNA copies in these two faeces samples was estimated to 2 372 and 11 880, respectively. However, we were unable to cultivate *C*. *burnetii* from faeces samples of infected ticks (*n* = 10), suggesting that they are not infectious.

### Transmission of *Coxiella burnetii* through tick bites

**Fig 2:**
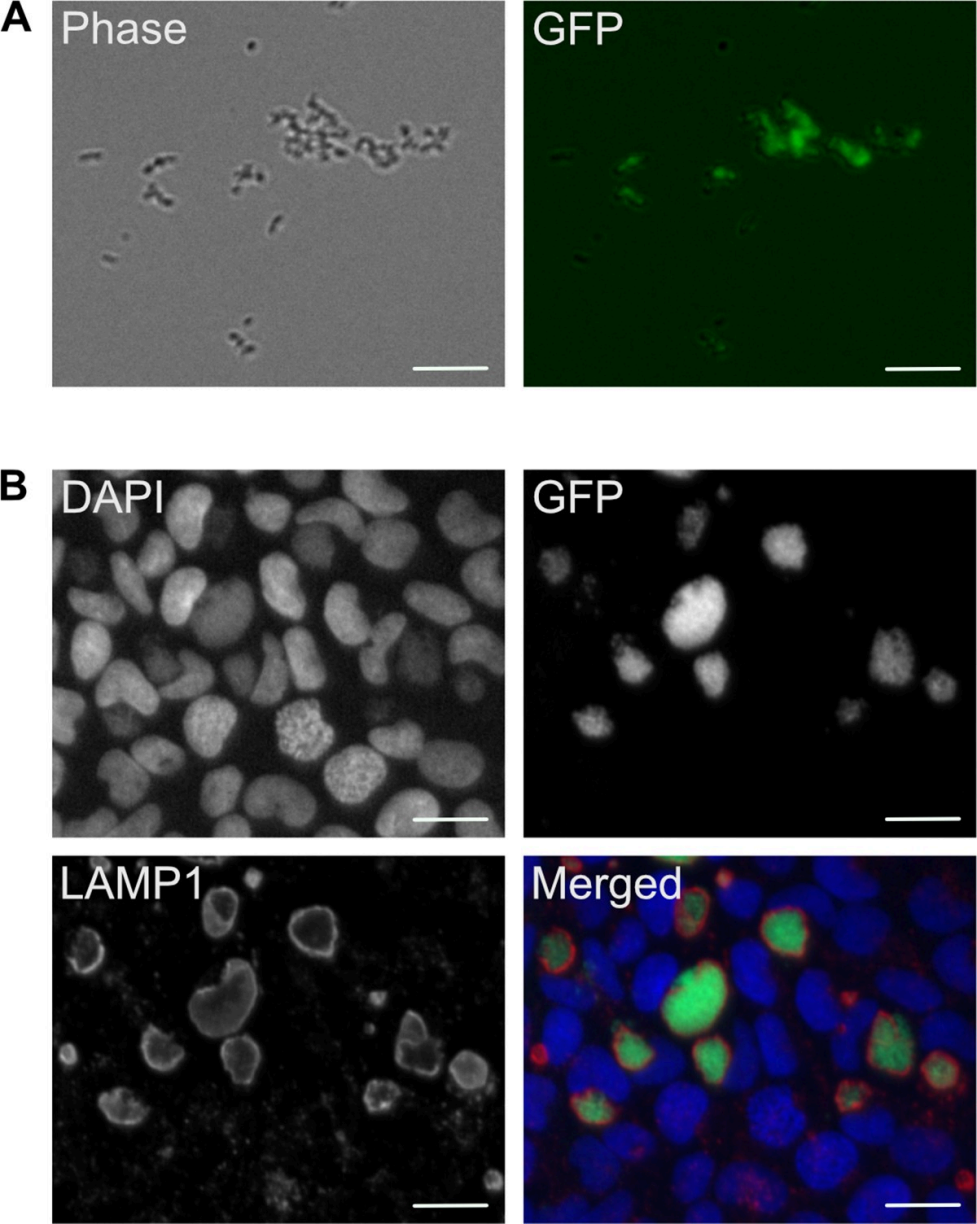
**A.** eGFP-expressing *C*. *burnetii* amplified from ACCM2 culture broth inoculated with samples from the second blood meal on which infected ticks fed. **B.** U2OS cells challenged with eGFP-expressing *C*. *burnetii* amplified as described in A were fixed and processed for immunofluorescence 6 days post-infection. Scale bars are 20 μm (A) and 10 μm (B).

The efficiency of transmission of *C*. *burnetii* through tick bites was evaluated 7 weeks after the initial feeding. To this end, an additional blood meal without *C*. *burnetii* was provided to adults of the infected batch (*n* = 8) and of the control batch (*n* = 4). qPCR validated the absence of *C*. *burnetii* DNA in the blood prior to the experiment. Following the experiment, qPCR did not reveal the presence of *C*. *burnetii* in the blood of feeding units on which infected and control ticks fed. However, when all blood samples on which infected ticks fed were used to inoculate ACCM2, viable eGFP-expressing *C*. *burnetii* were readily amplified (Fig 2A). By contrast, no positive culture was obtained from ACCM2 inoculated with blood on which control ticks fed. Importantly, eGFP-expressing *C*. *burnetii* isolated from blood samples on which infected ticks fed were effectively internalized by U2OS human epithelial cells upon infection, and developed the typical, LAMP1-positive intracellular replicative vacuole (Fig 2B).

### Transovarial transmission of *Coxiella burnetii* in ticks

Ten weeks after the exposure to *C*. *burnetii*, four gravid females of the infected batch laid eggs that we collected to formally assess the efficiency of *C*. *burnetii* transovarial transmission. Examination of the four females through qPCR showed that they were all positive for *C*. *burnetii* DNA. We further examined pools of 20 eggs per clutch (four pools, 80 eggs in total), and further screened for *C*. *burnetii* DNA by qPCR. No egg pool was found positive for infection. Four additional egg pools (80 eggs in total) were also used in axenic and cell cultures, but none was positive for *C*. *burnetii*.

## Discussion

In this study, we re-evaluated the transmission of *C*. *burnetii* in ticks, using the African soft tick *O*. *moubata* as model species. Observations of the three major traits related to vector competence show a very efficient transmission of *C*. *burnetii* by *O*. *moubata*: (i) The exposure to an infected blood meal consistently resulted in viable infection in all tick specimens examined. Infections remain detectable at least 10 weeks after the blood meal, showing that ticks can maintain *C*. *burnetii* over long periods. (ii) A trans-stadial transmission of infection from nymphs to adults was observed in all infected specimens. (iii) *O*. *moubata* retained the ability to transmit infectious *C*. *burnetii* when feeding. Altogether, this corroborates an early observation of *C*. *burnetii* transmission by *O*. *moubata* based on microscopy [20]. However, qPCR revealed that infection was not systemic, with substantial differences between tick organs. While adult female ticks were infected, we also did not observe *C*. *burnetii* in eggs, suggesting that transovarial transmission is not efficient. Finally, both qPCR and culture assays showed that excretion in faeces is not an efficient mode of transmission in *O*. *moubata*

Further observations showed that *C*. *burnetii* did not impact tick survival and molting to adulthood. This tolerance to infection may thus enhance the transmission risk by ticks over a large part of their lifespan. However, this tolerance to infection is actually not widespread in arthropods, since the avirulent Nine Mile phase II strain of *C*. *burnetii* (the same strain used in the present study) is also known to efficiently kill larvae of the greater wax moth, *G*. *mellonella*, in only a few days [36]. The *G*. *mellonella* moth is not a natural vector of *C*. *burnetii* but it is now used as an alternative model of infection: Infected specimens exhibit very similar cellular symptoms to those observed in infected vertebrates [36]. The fact that ticks and not *G*. *mellonella* are tolerant to *C*. *burnetii* may highlight pivotal biological differences between these arthropods. Indeed, recent observations showed that intracellular tick-borne pathogens modulate tick physiology and tick cell processes, including immunity and apoptosis, leading to tolerance of intracellular infections [41]. A similar modulation of *O*. *moubata* metabolism by *C*. *burnetii* may thus take place, not impacting tick survival and subsequently enhancing their vector competence. Obviously, more tick species are competent vectors of Q fever, as recently confirmed with two hard tick species of major medical and veterinary importance in Western Europe, *I*. *ricinus* and *D*. *marginatus* [32]. Of all the tick species examined thus far, only two, the soft ticks *O*. *gurneyi* and *O*. *turicata*, have been experimentally shown to be incompetent vectors [42,43]. Consequently, diverse tick species could be competent vectors for *C*. *burnetii*, at least in experimental systems.

Examination of infected *O*. *moubata* specimens showed that infection was not systemic: *C*. *burnetii* was largely detected in the midgut, to a lesser extent in the salivary glands and in the body, but only occasionally in the Malpighian tubules and reproductive organs. This pattern revealed that *C*. *burnetii* can cross the epithelium gut barrier and further colonize different tick organs. However, such infection pattern radically differs from early microscopy observations that consistently reported on systemic infections with high densities in Malpighian tubules and ovaries (reviewed in [15]). These contrasting results can be explained if endosymbionts of ticks, such as *Coxiella*-LE and *Francisella*-LE, were misidentified as *C*. *burnetii*. These endosymbionts are remarkably abundant in two organs of ticks: ovaries, which is consistent with their vertical transmission into developing oocytes, and Malpighian tubules, where B vitamins are possibly synthesized [35,44–46]. Since these endosymbionts of ticks are also intracellular with a roughly similar morphology to *C*. *burnetii*, the potential of misidentification is high without the use of molecular markers. Similar findings may explain why we did not observe any evidence of transovarial transmission whereas early microscopy studies did: Endosymbionts are maternally inherited, often in >90% of the progeny, and, as such, abundant in tick eggs [35,44–46] where they may have been misidentified as *C*. *burnetii*. To date, there is no molecular evidence of *C*. *burnetii* transovarial transmission in ticks: This suggests that maintenance of *C*. *burnetii* in tick populations cannot be possible without infectious transmission from vertebrates.

The most significant mechanism of *C*. *burnetii* transmission by *O*. *moubata* is salivary excretion during feeding, as typically observed for many other intracellular tick-borne pathogens. The detection of *C*. *burnetii* in salivary glands of some *O*. *moubata* specimens, and the isolation of viable *C*. *burnetii* from blood on which infected ticks fed, corroborate this hypothesis. However, the mechanism of transmission seems variable between tick species. Early microscopy studies found that ticks excrete large numbers of *C*. *burnetii* in faeces (up to 10^10^ bacteria per gram of faeces [17]); however, as discussed above, these bacteria could instead be endosymbionts. Our molecular assays detected *C*. *burnetii* DNA only in few faeces samples, and when present, the number of *C*. *burnetii* DNA copies was relatively low. Subsequent culture surveys of *O*. *moubata* faeces also failed to amplify *C*. *burnetii*, suggesting it is not a significant mechanism of transmission in this tick species and that only circulating *C*. *burnetii* DNA are excreted through faeces. Conversely, transmission through faeces (and not through biting) is efficient by *I*. *ricinus* and *D*. *marginatus*, as shown with the excretion of infectious *C*. *burnetii* in this way [32]. Biological dissimilarities between tick species could explain such variations in transmission mechanism: Indeed, soft ticks (Argasidae: *O*. *moubata*) and hard ticks (Ixodidae: *I*. *ricinus*, *D*. *marginatus*) differ in their feeding and digestive features [47]. Soft ticks are rapid feeders, ingesting a moderate amount of blood and further digesting only a portion of the blood meal as needed. This probably contributes to their ability to live for relatively long periods without an additional blood meal [47]. Hard ticks are slow feeders, ingesting a very large amount of blood and further digesting the entire blood meal. These biological dissimilarities may thus explain why *I*. *ricinus* and *D*. *marginatus*, and not *O*. *moubata*, excrete *C*. *burnetii* in their faeces.

As we used a phase II *C*. *burnetii* clone, some comment on the true infectious risk of these bacteria is appropriate. Given that phase II *C*. *burnetii* is an attenuated virulent form [37], it is likely that the infectious behaviour of isogenic phase I and phase II bacteria could be different in ticks. However, phase II *C*. *burnetii* is also internalized by host cells and replicate then with similar kinetics than phase I [37], and this explain why we observed a transmission of viable phase II *C*. *burnetii* able to infected human cells in laboratory conditions. Importantly, phase II *C*. *burnetii* is not avirulent: larvae of the *G*. *mellonella* moth are susceptible to phase II *C*. *burnetii* infections, as they showed typical cellular symptoms and died rapidly [36]. That ticks, and not the *G*. *mellonella* moth, are tolerant to infections showed that virulence in arthropods is not associated with *C*. *burnetii* phases. Furthermore, our investigations on phase II *C*. *burnetii* in *O*. *moubata* may also explain some differences with previous studies that only used phase I *C*. *burnetii*. However, Köner et *al*. [32] also used a phase II *C*. *burnetii* clone in *I*. *ricinus* and *D*. *marginatus* and observed a transmission through faeces that we did not observed in *O*. *moubata*. This showed that *C*. *burnetii* phases cannot explain all the differences reported by previous studies.

This study provides evidence that the African soft tick *O*. *moubata* is a competent vector of *C*. *burnetii*. However, Q fever is probably far more frequently transmitted through the airborne route than through ticks. Field surveys consistently showed that vector capacity (i.e. the transmission potential of a vector population in field conditions) of ticks in the field remains low: Only few unambiguous cases of natural *C*. *burnetii* infections in ticks exist [6,31]. Nevertheless, ticks may act as major drivers of the transmission and spatial dispersal of Q fever among vertebrates: Ticks can parasitize a broad diversity of hosts that potentially disperse over large distances on them. Ticks can also survive several months, or years depending on the species, off their vertebrate hosts and thus constitute substantial reservoirs of *C*. *burnetii* infections across time. Several pivotal traits of *C*. *burnetii* infections in ticks remain to be investigated; this includes the potential of *C*. *burnetii* to modulate tick metabolism and to enhance its tolerance, or the mechanisms driving variations of transmission between tick species. In this context, comparative taxonomic approaches will be highly valuable in enhancing our understanding of the epidemiology of Q fever in ticks.
